# Antimicrobial studies of unsymmetrical *bis*-1,2,3-triazoles

**DOI:** 10.1186/2191-2858-2-13

**Published:** 2012-04-04

**Authors:** Abid H Banday, Shameem A Shameem, Bashir A Ganai

**Affiliations:** 1 Department of Chemistry, Islamia College of Science and Commerce, Srinagar 190009, J&K, India; 2 Department of Biochemistry, University of Kashmir, Srinagar 190009, J&K, India

**Keywords:** azides, *bis*-triazoles, antimicrobials, microbial strains

## Abstract

Aryl azides were treated with allenylmagnesium bromide to generate 1,5-disubstituted butynyl 1,2,3-triazoles in a domino fashion, which upon Cu(I) catalyzed 1,3-dipolar cycloaddition with aryl azides afforded novel *bis*-1,2,3-triazoles in quantitative yields. The final products were analyzed for their antimicrobial activities against a panel of bacterial and fungal strains which revealed the products to be potent antimicrobials.

## 1. Introduction

Most of the nitrogen-containing molecules are pharmacologically very active which can be attributed to the fact that nitrogenous compounds are part and parcel of the biomolecular diversity [1-7]. Amongst the pharmacologically active nitrogenous compounds, a large number of 1,2,3-triazoles and their derivatives attracted considerable attention for the past few decades due to their chemotherapeutical value. Many 1,2,3-triazoles, including bis-triazoles, are found to be potent antimicrobial, analgesic, anti-inflammatory, local anesthetic, anti-convulsant, anti-neoplastic, anti-malarial, and anti-viral agents [8-10]. Some of them exhibited anti-proliferative, anticancer activity, and several are used as DNA cleaving agents and potassium channel activators. Such type of diverse biological functions is also reported with a variety of *bis*-triazoles. The 'click chemistry' approach has been the most widely used method for the synthesis of libraries of a large number of biologically active molecular frameworks particularly for the regioselective synthesis of 1,2,3-triazoles, which involves the copper(I)-catalyzed cycloaddition reaction between azides and terminal alkynes (CuAAC). This reaction has been termed as the 'cream of the crop' of 'click reactions' and has found application in various facets of drug discovery as it enables a modular approach to generate novel pharmacophores utilizing a collection of reliable chemical reactions [11,12]. Thus, the development of the copper(I)-catalyzed 'triazole click chemistry' has led to many interesting applications including the synthesis, medicinal chemistry, molecular biology, and material science. The bioorthogonality of azide and alkynes [13] has allowed the use of their [3 + 2] cycloaddition in various biological applications including target guided synthesis [14] and activity-based protein profiling [15]. Of particular interest would be the dimeric heterocycle-based ligands which are designed for specific target interactions. Various approaches reported for the synthesis of biologically relevant *bis*-triazoles include Cu(I)-catalyzed 1,3-dipolar cycloaddition of monoazides with diacetylenes or that of monoacetylenes with diazides. For example, the synthesis of *bis*-triazoles is reported by the reactions of *bis*(azidomethyl)benzenes with several substituted acetylenes [16]. Recently, much attention has been paid toward the synthesis and pharmacological evaluation of triazoles and *bis*-triazoles as potent HIV-1 protease inhibitors [17,18] and size-specific ligands for mRNA Hairpin loops [19], respectively. Keeping into consideration the tremendous biological potence of triazoles and *bis*-triazoles in general and the antimicrobial activity in particular, we, in our continuous endeavor toward the synthesis of pharmacologically active molecules, designed the synthesis of novel unsymmetrical *bis*-1,2,3-triazoles and then evaluated them for antimicrobial activities. The biological results obtained were very interesting and revealed most of the synthesized molecules to be potent antimicrobials.

## 2. Experimental

### 2.1. General methods

Melting points were recorded on Buchi Melting point apparatus D-545; IR spectra (KBr disks) were recorded on Bruker Vector 22 instrument. NMR spectra were recorded on Bruker DPX200 instrument in CDCl_3 _with TMS as internal standard for protons and solvent signals as internal standard for carbon spectra. Chemical shift values are mentioned in δ (ppm) and coupling constants are given in Hz. Mass spectra were recorded on EIMS (Shimadzu) and ESI-esquire 3000 Bruker Daltonics instrument. The progress of all reactions was monitored by TLC on 2 × 5 cm pre-coated silica gel 60 F254 plates of thickness of 0.25 mm (Merck). The chromatograms were visualized under UV 254-366 nm and iodine.

#### 2.2.1. Chemical synthesis

##### 2.2.1.1. General procedure for the synthesis of bis-1,2,3-triazoles (5)

**Scheme 1:**
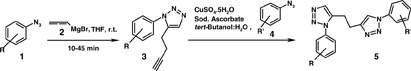
**Domino-click method for the synthesis of unsymmetrical bis 1,2,3-Triazoles**.

**Scheme 2:**
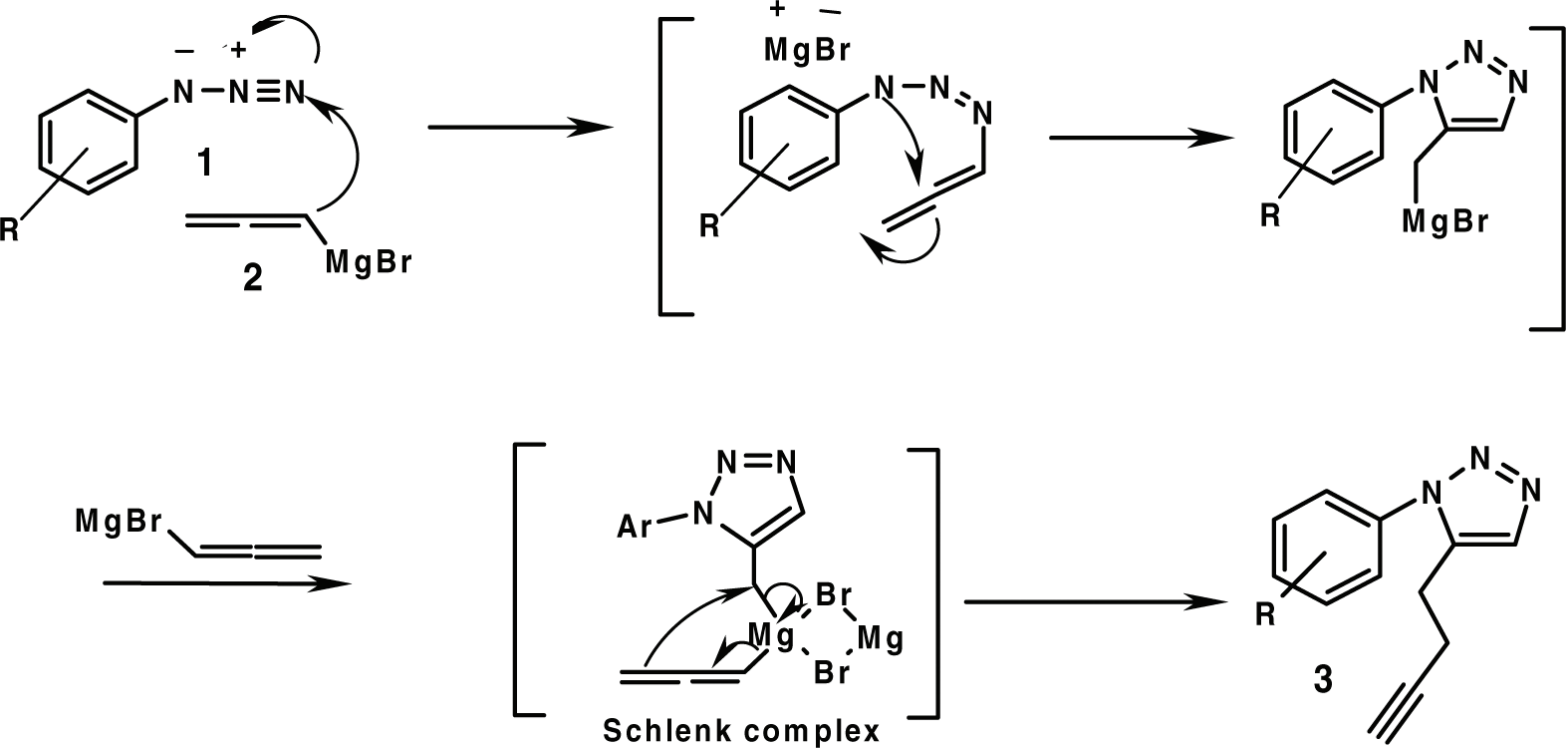
**Plausible mechanism for the formation of 5-butynyl triazoles 3**.

To a suspension of Mg turnings (1.6 g, 0.66 mol, 10 equiv.) in specially dried THF with HgCl_2 _(5 mg, 1% w/w of propargyl bromide) was added propargyl bromide (3.05 ml of an 80% wt. soln. in toluene, 4 mmol, 5 equiv.) in small portions while stirring the mixture at r.t. (*Note*: A small grain of HgCl_2 _is generally required to promote formation of the reagent.) The mixture was stirred at r.t. for 2 h to give a cloudy light green solution. The allenylmagnesium bromide generated as above was cooled to 0°-5° and added dropwise to a solution of 3-methylphenyl azide (1 g, 0.007 mol) maintaining the temperature between 0 and 5°C. The mixture was allowed to attain r.t., and stirring was continued at ambient temperature for 30 min, followed by quenching with aq. NH_4_Cl solution (10 mL) and diluting with AcOEt (50 mL). The org. layer was separated and the aq. layer extracted with AcOEt (2 × 20 mL). The combined org. layers were dried (anh. Na_2_SO_4_) and evaporated under reduced pressure to afford crude product, which was subjected to chromatography (silica gel, 60-120 mesh, elution; hexane/AcOEt gradient) to afford pure 5-(But-3-yn-1-yl)-1-(3-methylphenyl)-1H-1,2,3-triazoles 3 as a colorless liquid. 3-Methyl butynyl triazole (10 mmol) was stirred in 5 mL of *tert*-butanol and H_2_O (1:1 mixture). CuSO_4 _(12 mmol) and sodium ascorbate (50 mmol) were charged into the reaction mixture. After 15 min, 3-methylphenyl azide (10 mmol) was added to the above mixture, and stirred for 8 h. The mixture was diluted with AcOEt, the org. layer was separated, and the aq. layer extracted with AcOEt (2 × 20 mL). The combined org. layers were dried (anh. Na_2_SO_4_) and evaporated under reduced pressure to afford crude product 5 (Scheme 1), which was subjected to precipitation in hexane--AcOEt, affording pure *bis*-triazole 5 as an amorphous brown solid (only entries 2 and 13, see Table 1) (Scheme 2).

**Table 1:** *Bis*-triazoles prepared by 'Domino-Click' reaction

Entry	Triazole	Azide	Bis-triazole^a^	Yield (%)
a				92
b				91^b ^
c				89
d				91
e				88
f				92
g				90
h				90
i				91
j				93
k				92
l				89
m				90^c ^

The analytical data of all the isolated *bis*-triazoles is given as under.

###### 1-(4-methoxyphenyl)-5-(2-(1-*m*-tolyl-1H-1,2,3-triazol-4-yl)ethyl)-1H-1,2,3-triazole (5a)

Syrupy brownish liquid. IR (KBr) cm^-1^: 3453, 2913, 2865, 1593, 1212, 1080, and 685; ^1^H NMR (CDCl_3_): δ 2.44 (s, 3H); 3.12 (m, 4H); 3.87 (s, 3H); 7.02 (d, 2H, *J *= 8.89 Hz); 7.35 (m, 6H); 7.50 (s, 1H), 7.60 (s, 1H); ^13^C NMR (500 MHz, CDCl_3_): δ 21.40, 23.44, 24.53, 55.63, 114.74, 117.53, 119.41, 121.14, 126.71, 128.00, 129.53, 138.11, 140.03, 148.22, 161.12.; ESI-MS: 383 (M^+ ^+ Na); Anal. Calcd. for C_20_H_20_N_6_O: C, 66.65; H, 5.59; N, 23.32; Found: C, 66.83; H, 5.38; N, 23.51.

###### 4-(4-(2-(3-(4-methoxyphenyl)-3H-1,2,3-triazol-4-yl)ethyl)-1H-1,2,3-triazol-1-yl)benz-oic acid (5b)

Amorphous white solid. m. p. 195-197°C; IR (KBr) cm^-1^: 3413, 2922, 2860, 1593, 1234, 1017, and 690; ^1^H NMR (CDCl3): δ δ 3.10 (t, 2H, *J *= 5.97); 3.12 (t, 2H, *J *= 5.97); 3.84 (s, 3H); 7.08 (d, 2H, *J *= 8.90 Hz); 7.37 (d, 2H, *J *= 8.90 Hz); 7.72 (s,1H), 7.93 (d, 2H, *J *= 8.63 Hz), 8.21 (d, 2H, *J *= 8.90 Hz); 8.33 (s, 1H); ^13^C NMR (500 MHz, CDCl3): δ 22.50, 23.59, 54.45, 114.15, 115.43, 119.17, 119.21, 120.13, 126.36, 127.43, 130.78, 131.06, 147.23, 200.12; ESI-MS: 391 (M^+ ^+ H); Anal. Calcd. for C_20_H_18_N_6_O_3_: C, 61.53; H, 4.65; N, 21.53; Found: C, 61.71; H, 4.82; N, 21.79.

###### 1-(4-methoxyphenyl)-5-(2-(1-(3-nitrophenyl)-1H-1,2,3-triazol-4-yl)ethyl)-1H-1,2,3-triazole (5c)

Syrupy grayish semisolid. IR (KBr) cm^-1^: 3393, 2897, 2867, 1582, 1244, 1018, and 689; ^1^H NMR (CDCl3): δ 3.10 (t, 2H, *J *= 5.78); 3.23 (t, 2H, *J *= 5.78); 3.85 (s, 3H); 7.05 (d, 2H, *J *= 8.94 Hz); 7.38 (d, 2H, *J *= 8.94 Hz); 7.73-7.88 (m, 2H); 8.22-8.32 (m, 2H); 8.42 (s, 1H); 8.68 (m, 1H); ^13^C NMR (500 MHz, CDCl3): δ 22.20, 23.69, 55.45, 113.15, 116.43, 118.17, 119.41, 120.13, 127.36, 127.43, 130.78, 132.06, 142.33, 148.25; ESI-MS: 392 (M^+ ^+ H); Anal. Calcd. for C_19_H_17_N_7_O_3_: C, 58.31; H, 4.38; N, 25.05; Found: C, 58.54; H, 4.5; N, 25.21.

###### 1-(4-methoxyphenyl)-5-(2-(1-*p*-tolyl-1H-1,2,3-triazol-4-yl)ethyl)-1H-1,2,3-triazole (5d)

Syrupy grayish semisolid. IR (KBr) cm^-1^: 3423, 2932, 2876, 1593, 1234, 10179, and 694; ^1^H NMR (CDCl_3_): δ 2.45 (*s*, 3H); 3.15 (m, 4H); 3.87 (s, 3H); 7.02 (d, 2H, *J *= 8.89 Hz); 7.18 (d, 2H, *J *= 8.89 Hz), 7.37 (d, 2H, *J *= 8.90 Hz); 7.47 (d, 2H, *J *= 8.90 Hz); 7.50 (s,1H), 7.80 (s, 1H); ^13^C NMR (500 MHz, CDCl_3_): δ 18.50, 22.23, 23.59, 54.45, 114.15, 115.43, 119.17, 119.51, 120.13, 126.36, 127.33, 130.78, 135.06, 147.23; ESI-MS: 383 (M^+ ^+ Na); Anal. Calcd. for C_20_H_20_N_6_O: C, 66.65; H, 5.59; N, 23.32; Found: C, 66.42; H, 5.68; N, 23.54.

###### 1-(3-chlorophenyl)-5-(2-(1-*p*-nitrophenyl-1H-1,2,3-triazol-4-yl)ethyl)-1H-1,2,3-triazole (5e)

Syrupy grayish semisolid. IR (KBr) cm^-1^: 3411, 2945, 2898, 1597, 1265, 1067, and 702; ^1^H NMR (CDCl_3_): δ 3.17-3.40 (m, 4H); 7.34-7.57 (m, 2H); 7.77 (m, 2H), 7.86 (s, 1H); 8.17 (d, 2H, *J *= 7.85 Hz); 8.30 (d, 2H, *J *= 7.85), 8.55 (s, 1H); ^13^C NMR (500 MHz, CDCl_3_): δ 19.25, 21.23, 23.56, 114.15, 115.43, 119.17, 119.51, 120.13, 126.36, 128.33, 130.87, 136.06, 146.23; ESI-MS: 418 (M^+ ^+ Na); Anal. Calcd. for C_18_H_14_ClN_7_O: C, 54.62; H, 3.57; N, 24.77; Found: C, 54.85; H, 3.38; N, 24.86.

###### 1-(3-nitrophenyl)-5-(2-(1-*m*-tolyl-1H-1,2,3-triazol-4-yl)ethyl)-1H-1,2,3-triazole (5f)

Syrupy brownish semisolid. IR (KBr) cm^-1^: 3417, 2954, 2856, 1587, 1235, 1079, and 698; ^1^H NMR (CDCl3): δ 2.44 (s, 3H); 3.17-3.25 (m, 4H); 7.39 (d, 2H, *J *= 7.40 Hz); 7.50 (s, 2H), 7.70-7.82 (m, 4H); 8.36 (m, 2H); ^13^C NMR (500 MHz, CDCl3): δ 19.54, 21.23, 23.59, 114.15, 116.41, 119.17, 119.51, 120.13, 128.32, 127.35, 130.88, 136.06, 146.23, 153.22.; ESI-MS: 398 (M^+ ^+ Na); Anal. Calcd. for C_19_H_17_N_7_O_2_: C, 60.79; H, 4.56; N, 26.12; Found: C, 60.95; H, 4.72; N, 26.34.

###### 1-(3-nitrophenyl)-5-(2-(1-(3-nitrophenyl)-1H-1,2,3-triazol-4-yl)ethyl)-1H-1,2,3-triazole (5g)

Syrupy brownish semisolid. IR (KBr) cm^-1^: 3419, 2965, 2857, 1580, 1265, 1099, and 714; ^1^H NMR (CDCl3): δ 3.21 (t, 2H, *J *= 5.44); 3.30 (t, 2H, *J *= 5.44); 7.79 (m, 4H, *J *= 7.40 Hz); 7.87 (s, 1H), 8.15 (d, 2H, *J *= 7.82 Hz); 8.38 (m, 2H), 8.54 (s, 1H); ^13^C NMR (500 MHz, CDCl3): δ 19.47, 21.34, 22.89, 114.15, 116.67, 119.77, 118.52, 120.13, 127.32, 128.35, 130.32, 136.18, 146.56, 154.31; ESI-MS: 407 (M^+ ^+ H); Anal. Calcd. for C_18_H_14_N_8_O_4_: C, 53.20; H, 3.47; N, 27.57; Found: C, 53.11; H, 3.62; N, 27.34.

###### 5-(2-(1-(3-nitrophenyl)-1H-1,2,3-triazol-4-yl)ethyl)-1-*o*-tolyl-1H-1,2,3-triazole (5h)

Syrupy brownish semisolid. IR (KBr) cm^-1^: 3418, 2954, 2856, 1587, 1235, 1079, and 698; ^1^H NMR (CDCl3): δ 2.36 (s, 3H); 3.09 (m, 4H); 7.22 (m, 2H); 7.43 (m, 4H), 7.68 (s, 1H); 7.71-7.84 (m, 2H), 8.17 (d, 1H, *J *= 7.66); ^13^C NMR (500 MHz, CDCl3): δ 18.54, 21.23, 23.59, 114.15, 117.41, 119.17, 119.51, 120.13, 128.32, 127.35, 130.88, 136.06, 146.23, and 155.23; ESI-MS: 398 (M^+ ^+ Na); Anal. Calcd. for C_19_H_17_N_7_O_2_: C, 60.79; H, 4.56; N, 26.12; Found: C, 60.95; H, 4.72; N, 26.34.

###### 1-*o*-tolyl-5-(2-(1-*m*-tolyl-1H-1,2,3-triazol-4-yl)ethyl)-1H-1,2,3-triazole (5i)

Syrupy brownish semisolid. IR (KBr) cm^-1^: 3417, 2944, 2856, 1587, 1235, 1079, and 695; ^1^H NMR (CDCl3): δ 2.07 (s, 3H); 2.15 (s, 3H); 3.12 (m, 2H); 7.54 (m, 2H); 7.63 (s, 1H); 7.74 (m, 3H), 8.08-8.23 (m, 3H); 8.57 (s,1H); ^13^C NMR (500 MHz, CDCl3): δ 19.68, 19.19, 21.58, 23.38, 116.10, 121.14, 122.22, 124.7, 129.82, 130.30, 131.31, 132.0, 135.80, 136.80, 137.83, 140.01, 148.20; ESI-MS: 367 (M^+ ^+ Na); Anal. Calcd. for C_20_H_20_N_6_: C, 69.75; H, 5.85; N, 24.40; Found: C, 69.90; H, 5.52; N, 24.61.

###### 5-(2-(1-(2-nitrophenyl)-1H-1,2,3-triazol-4-yl)ethyl)-1-*p*-tolyl-1H-1,2,3-triazole (5j)

Syrupy greyish semisolid. IR (KBr) cm^-1^: 3427, 2966, 2865, 1576, 1235, 1079, and 687; ^1^H NMR (CDCl_3_): δ 2.45 (s, 3H); 3.21-3.23 (m, 4H); 7.38-7.45 (d, 2H, *J *= 8.00 Hz); 7.70 (d, 2H, *J *= 8.00 Hz), 7.81 (m, 1H); 7.90 (m, 3H), 8.14 (m, 2H); ^13^C NMR (500 MHz, CDCl_3_): δ 19.85, 23.00, 23.54, 125.19, 125.27, 127.31, 129.71, 129.97, 130.89, 133.80, 134.97, 140.39, 144.60; ESI-MS: 367 (M^+ ^+ Na); Anal. Calcd. for C_19_H_17_N_7_O_2_: C, 60.79; H, 4.56; N, 26.12; Found: C, 60.61; H, 4.72; N, 26.29.

###### 5-(2-(1-(3-nitrophenyl)-1H-1,2,3-triazol-4-yl)ethyl)-1-*p*-tolyl-1H-1,2,3-triazole (5k)

Syrupy greyish semisolid. IR (KBr) cm^-1^: 3417, 2986, 2865, 1576, 1233, 1089, and 677; ^1^H NMR (CDCl_3_): δ 2.45 (s, 3H); 3.08-3.22 (m, 4H); 7.33 (s, 4H); 7.50 (s, 1H), 7.52-7.72 (m, 4H), 7.89 (d, 1H, *J *= 7.89 Hz); ^13^C NMR (500 MHz, CDCl_3_): δ 15.82, 21.24, 23.33, 118.90, 122.73, 125.56, 130.23, 132.51, 133.69, 133.80, 135.61, 136.81, 139.97, 142.41, 144.43, 151.16; ESI-MS: 398 (M^+ ^+ Na); Anal. Calcd. for C_19_H_17_N_7_O_2_: C, 60.79; H, 4.56; N, 26.12; Found: C, 60.92; H, 4.30; N, 26.26.

###### 1-*m*-tolyl-5-(2-(1-*m*-tolyl-1H-1,2,3-triazol-4-yl)ethyl)-1H-1,2,3-triazol (5l)

Amorphous brown solid; m.p. 175°C. IR (KBr) cm^-1^: 3429, 3138, 2922, 2860, 1612, 1593, 1549, 1494, 1383, 1234, 1165, 1089, 1047, 1017, 980, 873, 849, 786, 690, and 618; ^1^H NMR (CDCl3): 2.33 (s, 3H); 2.43 (s, 3H); 3.05 (t, 2H, *J *= 6.2 Hz); 3.20 (t, 2H, *J *= 6.2 Hz); 7.25-7.59 (m, 8H); 7.73 (s, 1H); 8.36 (s, 1H). ^13^C NMR (500 MHz, CDCl3): 19.8, 19.9, 22.8, 23.8, 117.0, 120.4, 122.2, 125.7, 129.1, 129.8, 130.3, 132.0, 135.8, 136.8, 137.8, 140.0, 146.2. ESI-MS: 367 (M^+ ^+ Na). Anal. calc. for C_20_H_20_N_6 _: C, 69.75; H, 5.85; N, 24.40; Found: C, 69.80; H, 5.82; N 24.51.

#### 2.2.2. Biology

The bacterial strains used for the analysis were *Bacillus subtilis *(MTCC 619), *Staphylococcus epidermidis *(MTCC 435), *Proteus vulgaris *(MTCC 426), and *Pseudomonas aeruginosa *(MTCC 424). The fungal strains used were *Aspergillus niger *(MTCC 1344) and *Penicillium chrysogenum *(MTCC 947). All the bacterial and fungal strains were obtained from The Microbial Type Culture Collection and Gene Bank (MTCC), Institute of Microbial Technology (IMTECH), Chandigarh, India. Kenamycin and flucanazole were used as standard antibacterial and antifungal substances, respectively, under similar conditions for comparison. Dimethyl sulfoxide (DMSO) was used as negative control.

The test organisms were cultured on agar slants, incubated 24 h at 37 ± 0.5°C and 24-48 h at 27 ± 0.2°C for bacteria and fungi, respectively, to get the freshly prepared cultures. The steroidal derivatives were evaluated for antimicrobial activity against these freshly prepared strains of test organisms by agar diffusion method [20,21]. Muller Hinton Agar (MHA) and Potato Dextrose Agar (PDA) were used as nutrient media for bacterial and fungal strains, respectively. The media (MHA &PDA) were prepared using distilled water and 20 mL of it was transferred into 50-mL test tubes, the test tubes were tightly plugged with cotton and sterilized in autoclave at 15 lb/in^2 ^for 15 min as directed by the manufacturer. After sterilization, the medium was inoculated with freshly cultured bacterial strains under sterile condition, i.e., under Laminar Flow. The inoculation was done when the temperature of the medium reached 50-40°C, so that test organism may not die at higher temperature. The medium inoculated with test microorganisms was transferred into the plates of 90-mm size under sterile conditions. The medium was allowed to solidify and the wells (4/plate) of 6-mm-diameter and 50 μL volume were bored on it using sterile cork borer. The solution of test compound 1000 μg/mL was prepared in DMSO and the wells bored on the medium were each filled (50 μg) with test compound using micropipette (20-200 μL). Four wells were bored on the plates and each filled with same compound and two plates for each test compound were taken and the experiment was repeated twice. The disks of Kenamycin and Flucanazole were also incorporated into the medium for comparison (10-30 μg). The plates containing test organism and test material in contact were incubated at 37 ± 0.5°C for 24 h. Same procedure was employed for antifungal activity; however, the culture strains of fungi were maintained on PDA and spores were transferred into the PDA medium and the plates were incubated at 27 ± 0.2°C for 24-48 h. Inhibition of growth of test organisms (bacterial & fungal) in presence of test material and standard was measured with the help of standard scale and the mean values of inhibition zones are reported in Table 2.

**Table 2:** Antibacterial and antifungal screening data of compounds 5a-l

Compounds	Zone of inhibition (mm)					
	
	Antibacterial activities				Antifungal activities	
	
	*B. subtilis *(MTCC 619)	*S. epidermidis *(MTCC 435)	*P. Vulgaris *(MTCC 426)	*P. aeruginosa *(MTCC 424)	*A. niger *(MTCC1344)	*P. chrysogenum *(MTCC 947)
3a	08	09	10	12	-	10
3b	07	12	07	03	11	10
3c	-	11	10	12	10	10
3d	11	12	-	12	12	12
3e	12	10	13	10	10	16
3f	09	14	14	-	13	14
3g	12	-	08	11	12	09
3h	14	12	10	10	11	10
3i	10	12	-	09	13	12
3j	08	-	10	11	11	12
3k	07	16	11	11	12	13
3l	10	09	11	08	09	13
Control	-	-	-	-	-	-
Kenamycin	23	24	22	17	-	-
Flucanazole	-	-	-		18	14

*P. vulgaris, Proteus vulgaris*; *B. subtilis, Bacillus subtilis; S. epidermidis, Staphylococcus epidermidis*; *P. aeruginosa, Pseudomonas aeruginosa; *DMSO, negative control; well diameter/vol.- 6 mm/50 μl; Kenamycin, standard for antibacterial activity. Flucanazole, standard for anti fungal activity.

Table 2 gives the antimicrobial screening data obtained after treating different microbial strains with test doses of the different *bis*-triazolyl derivatives and the values are reported in terms of zone of inhibition in "mm".

It is clear from the above data that all the compounds **3a-l **showed significant antimicrobial activity against all microbial strains used for testing. It is evident from the data that even the position of substituent on the aromatic ring influences the relative activity which can be attributed to their differences in either the bioavailability or the protein-binding properties.

## 3. Results and discussions

The wide range of pharmacological activities especially the antimicrobial potential [14,22-27] of triazole and *bis*-triazole systems prompted us to design the synthesis of a library of unsymmetrical *bis*-1,2,3-triazoles based on a stepwise synthetic route involving domino addition of allenylmagnesium bromide to aryl azides resulting in a serendipitous formation of 5-butynylated triazoles in good yields (> 70%) instead of 4-butynylated triazoles. 5-Butynylated triazoles upon Cu(I) catalyzed 1,3-dipolar cycloaddition with aryl azides generated bis-1,2,3-triazoles in quantitative yields. The products together with the approach for their synthesis being novel, the intermediate 5-butynylated triazoles and the final product, the bistriazole, were characterized by IR, ^1^H/^13^C NMR, and mass spectral analysis. The intermediate 3 undergoes a high yielding regioselective Cu(I) catalyzed 1,3-dipolar cycloaddition with aryl azides (click reaction) to afford quantitative yields of the product, i.e., *bis*-1,2,3-triazoles, which were isolated in pure form after precipitation. The isolated products were evaluated for their antimicrobial activities against a panel of bacterial and fungal cell lines. The biological results were highly encouraging paving a way for the futuristic medicinal chemistry work based on these scaffolds.

## 4. Conclusion

In conclusion, we have developed an unprecedented, convenient strategy for the synthesis of novel, biologically important *bis*-1,2,3-triazoles employing a domino reaction followed by the copper catalyzed 'click' protocol. The products thus obtained were found to be potent antimicrobial agents.

## Competing interests

The authors declare that they have no competing interests.
